# Micro RNA-175 Targets *Claudin-1* to Inhibit Madin–Darby Canine Kidney Cell Adhesion

**DOI:** 10.3390/genes15101333

**Published:** 2024-10-16

**Authors:** Xiaoyun Li, Fangfang Ma, Siya Wang, Tian Tang, Liyuan Ma, Zilin Qiao, Zhongren Ma, Jiamin Wang, Zhenbin Liu

**Affiliations:** 1 Engineering Research Center of Key Technology and Industrialization of Cell-Based Vaccine, Ministry of Education, Northwest Minzu University, Lanzhou 730030, China; lixy000920@163.com (X.L.); fangf0111@163.com (F.M.); wsy19991216@163.com (S.W.); tiantang202203@163.com (T.T.); qiaozilin@xbmu.edu.cn (Z.Q.); mazhr@foxmail.com (Z.M.); 2 Gansu Tech Innovation Center of Animal Cell, Biomedical Research Center, Northwest Minzu University, Lanzhou 730030, China; 3 Life Science and Engineering College, Northwest Minzu University, Lanzhou 730030, China; 15596045224@163.com; 4 Key Laboratory of Biotechnology & Bioengineering of State Ethnic Affairs Commission, Biomedical Research Center, Northwest Minzu University, Lanzhou 730030, China

**Keywords:** MDCK, cell adhesion, CLDN1, miR-175, suspension

## Abstract

**Background:** The Madin–Darby canine kidney (MDCK) cell line constitutes a key component of influenza vaccine production, but its dependence on adherent growth limits cell culture density and hinders vaccine yield. There is evidence that the use of gene editing techniques to inhibit cell adhesion and establish an easily suspended cell line can improve vaccine yield; however, the mechanisms underlying MDCK cell adhesion are unclear. Methods: In this study, we used transcriptomics to analyse differentially expressed mRNAs and miRNAs in adherent and suspension cultures of MDCK cells. **Results**: We found that claudin-1 (CLDN1) expression was downregulated in the suspension MDCK cells and that CLDN1 promotes MDCK cell–extracellular matrix adhesion. Additionally, microRNA (miR)-175 expression was upregulated in the suspension MDCK cells. Importantly, we demonstrated that miR-175 inhibits MDCK cell adhesion by targeting the CLDN1 3′-untranslated region (UTR). These findings contribute to a more comprehensive understanding of the regulatory mechanisms modulating cell adhesion and provide a basis for establishing suspension-adapted, genetically engineered cell lines. Our work could also facilitate the identification of targets for tumour therapy.

## 1. Introduction

The Madin–Darby canine kidney (MDCK) cell line plays a critical role in influenza A and B vaccine production [1,2,3]. However, wild-type MDCK cells generally require adherent growth, constraining cell culture density and limiting vaccine yield. Although MDCK cells can be domesticated by direct and indirect methods, thereby inducing single-cell suspension cultures in specific cell culture media [4], these conventional suspension domestication methods are time-consuming and are likely to alter other biological properties. Alternatively, MDCK cells can be adapted to suspension growth in shake flasks through the exogenous transfection of the human *ST6GALNAC5* gene to inhibit cell adhesion [5]. Regardless of these techniques, the coding genes, non-coding genes, and regulatory mechanisms controlling cell adhesion in MDCK cells remain unclear.

There is increasing evidence that microRNAs (miRNAs), a subset of single-stranded, non-coding RNAs, regulate cell adhesion by binding to 3′-untranslated regions (UTRs) within mRNA. A single miRNA can regulate cell adhesion by targeting multiple cell adhesion molecules. For example, miR-200c-3p inhibits cell adhesion by synergistically targeting adhesion molecules, such as zinc finger E-box binding homeobox 1 (*ZEB1*) 3′ UTR and talin 1 (*TLN1*) 3′ UTR [6,7]. Conversely, multiple miRNAs can target and regulate the same cell adhesion molecule. miR-124 [8], miR-29 [9], miR-17 [10], miR-183-5p [11], and miR-134 [12] reportedly inhibit cell adhesion by directly targeting the integrin subunit β 1 (*ITGB1*) 3′-UTR. Furthermore, miRNAs can affect various pathological processes, such as tumour metastasis, angiogenesis, and inflammation [13,14], by regulating cell adhesion. In summary, miRNAs clearly influence cell adhesion by regulating target genes, but no studies have identified the miRNAs (or the potential target genes) that regulate MDCK cell adhesion.

Cell adhesion mainly consists of cell–cell adhesion and cell–extracellular matrix adhesion, both regulated by extracellular matrix proteins and cell-specific adhesion proteins (e.g., desmosome junction proteins, cadherins, and tight junction proteins). Claudin-1 (CLDN1), a tight junction protein that regulates epithelial cell permeability, is involved in biological processes such as cell adhesion, cell differentiation, and DNA damage repair; it has also been implicated in tumour formation [15,16,17]. CLDN1 regulates intercellular adhesion in various cell types, including cervical adenocarcinoma [18], colorectal cancer [19], and triple-negative breast cancer [20]. Most studies concerning the CLDN1-mediated regulation of cell adhesion have emphasised its effect on intercellular adhesion, whereas few have explored its effect on cell–extracellular matrix adhesion. To our knowledge, no studies have been conducted regarding the effect of CLDN1 on MDCK cell adhesion, and it is unclear whether miRNAs play a regulatory role in CLDN1 function.

Therefore, this study explored the miRNAs affecting MDCK cell adhesion and sought to identify their target genes. Using RNA-Seq technology, we analysed differentially expressed mRNAs and miRNAs in adherent and suspension MDCK cells, then examined the effects of their target genes on MDCK cell adhesion. We found that miR-175 inhibits MDCK cell–extracellular matrix adhesion by targeting the *CLDN1* 3′-UTR. These findings contribute to a more comprehensive understanding of animal cell adhesion mechanisms and offer potential targets for the establishment of suspension-adapted MDCK cell lines via genetic engineering. Additionally, considering that abnormalities in cell adhesion can promote tumour metastasis [21,22], the results of this study may provide novel targets for tumour therapy.

## 2. Results

### 2.1. The Transcriptomic Analysis of Differentially Expressed mRNAs and miRNAs in Suspension and Adherent MDCK Cells

**Figure 1:**
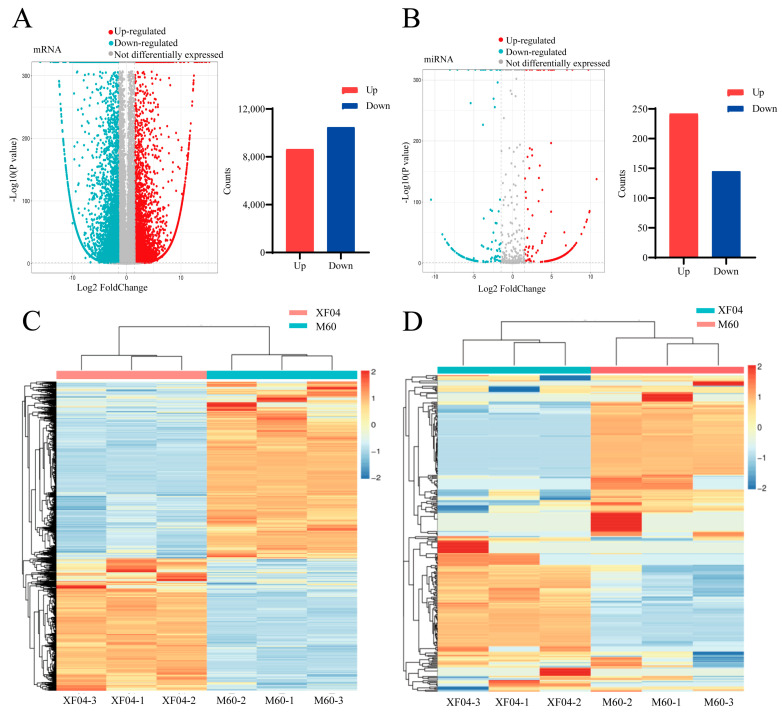
Volcano plot and heatmap of differentially expressed mRNAs and miRNAs in XF04 compared with M60. (**A**) mRNA volcano plot: Light green spots indicate downregulated expression, grey spots indicate not differentially expressed, and red spots indicate upregulated expression. Total number of mRNAs: red column indicates upregulated expression and blue column indicates downregulated expression. (**B**) miRNA volcano plot: Light green spots indicate downregulated expression, dark spots indicate not differentially expressed, and red spots indicate upregulated expression. Total number of miRNAs: red column indicates upregulated expression and blue column indicates downregulated expression. (**C**) mRNA heatmap: Each column represents a sample, and each row represents an mRNA. mRNA expression levels in different samples are represented by different colours. Red indicates higher expression, whereas blue indicates lower expression. Light green denotes M60, and pink denotes XF04. (**D**) miRNA heatmap: Each column represents a sample, and each row represents an miRNA. miRNA expression levels in different samples are represented by different colours. Red indicates higher expression, whereas blue indicates lower expression. Light green denotes M60 (the adherent MDCK cell), and pink denotes XF04 (the suspension MDCK cell).

To analyse the mRNA and miRNA expression profiles in adherent and suspension MDCK cells, we extracted total RNA from those cells and constructed cDNA libraries for Illumina sequencing. In total, we detected 50,603 differentially expressed mRNAs: 8633 were upregulated and 10,479 were downregulated in the suspension MDCK cells compared with the adherent MDCK cells (Figure 1A). Additionally, we identified 744 differentially expressed miRNAs: 242 were upregulated and 145 were downregulated in the suspension MDCK cells compared with the adherent MDCK cells (Figure 1B). Differentially expressed gene clustering heatmaps (Figure 1C,D) revealed significant differences in the mRNA and miRNA expression patterns between the adherent and the suspension MDCK cells, whereas the expression patterns were relatively stable and reproducible within each cell type. These results indicated a robust relationship between the differentially expressed genes and the observed differences in cell adhesion.

**Figure 2:**
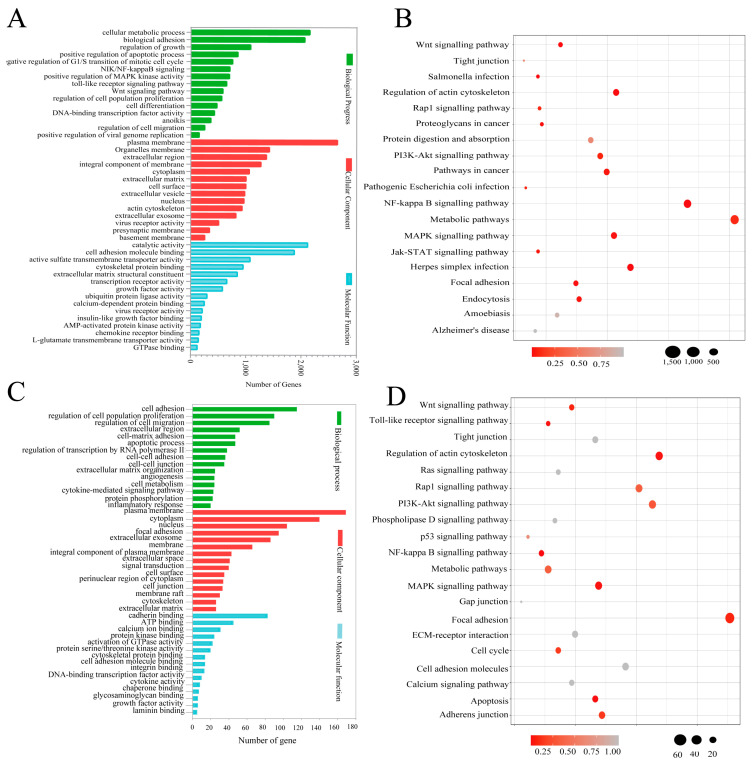
GO and KEGG functional analyses of all differentially expressed genes. (**A**) GO functional annotation histogram of all differentially expressed genes. GO terms were ranked according to number of genes across biological processes (green), cellular components (red), and molecular functions (blue). (**B**) KEGG pathway assessment of all differentially expressed genes. Lower *p*-values indicate greater pathway enrichment. Black circles represent gene numbers; a larger circle indicates a higher number of genes. (**C**) GO functional annotation histogram of adhesion-related differentially expressed mRNAs. GO terms were ranked according to number of genes across biological processes (green), cellular components (red), and molecular functions (blue). (**D**) KEGG pathway assessment of adhesion-related differentially expressed mRNAs. Lower *p*-values indicate greater pathway enrichment. Black circles represent gene numbers; a larger circle indicates a higher number of genes.

Gene Ontology (GO) analyses of the differentially expressed mRNA clusters showed that 2605 mRNAs were involved in cell adhesion. Moreover, differentially expressed mRNAs in the cellular component (CC) cluster were predominantly located in the plasma membrane, and mRNAs in the molecular function (MF) cluster involved in cellular adhesion molecule binding displayed differential expression between the two cell types (Figure 2A). The Kyoto Encyclopedia of Genes and Genomes (KEGG) pathway enrichment analysis showed the greatest enrichment in metabolism-related signalling pathways, as well as the nuclear factor (NF)-κB, mitogen-activated protein kinase (MAPK), and phosphoinositide 3-kinase (PI3K)–protein kinase B (Akt) signalling pathways (Figure 2B), suggesting that these signalling pathways are associated with altered cell adhesion properties. Of the 2605 differentially expressed mRNAs, we identified 743 that were specifically related to cell adhesion, on the basis of gene function annotations in the GO, KEGG, and UniProt databases. A GO analysis of the 743 differentially expressed mRNAs showed that the greatest enrichment in the biological process (BP) cluster involved cell adhesion processes, that cell adhesion-associated differentially expressed mRNAs in the CC cluster were mainly located in the plasma membrane, and that differentially expressed mRNAs in the MF cluster were mainly involved in calcineurin binding (Figure 2C). Additionally, a KEGG analysis of the 743 differentially expressed mRNAs enriched the top 20 most significant pathways, as shown in Figure 2D; the most enriched signalling pathway was focal adhesion, suggesting that these differentially expressed mRNAs regulate cell adhesion through the focal adhesion signalling pathway.

### 2.2. CLDN1 Expression Was Significantly Downregulated in the Suspension MDCK Cells

**Figure 3:**
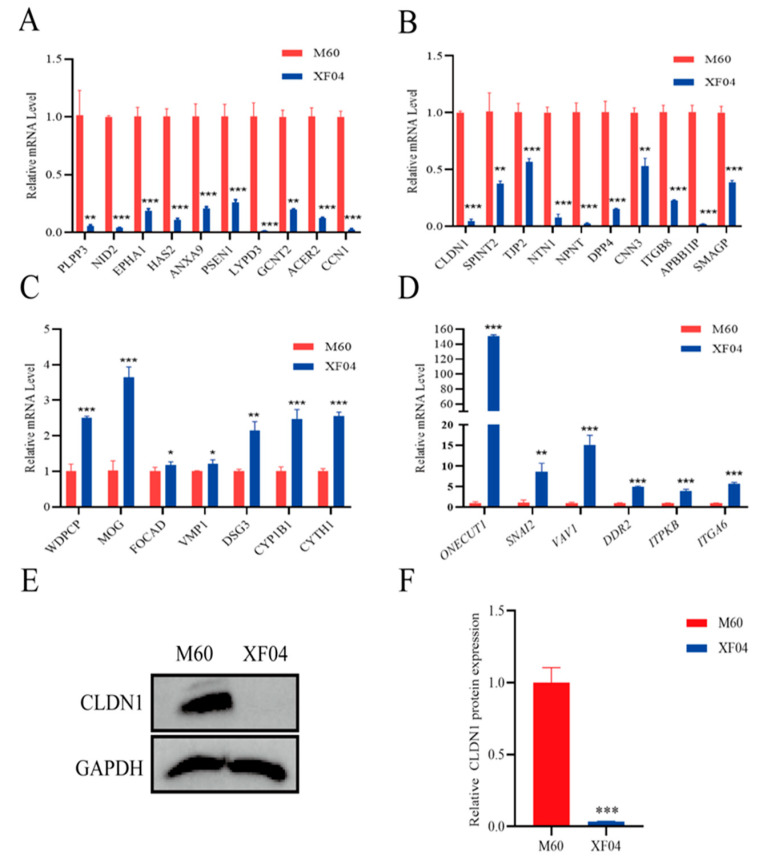
CLDN1 expression was downregulated in the suspension MDCK cells. A fluorescent reverse transcription–quantitative PCR was performed to detect the relative mRNA expression levels of adhesion-related genes identified via transcriptomics. (**A**,**B**) Genes with downregulated expression in XF04 (the suspension MDCK cell) compared with M60 (The adherent MDCK cell), and (**C**,**D**) genes with upregulated expression in XF04 compared with M60. (**E**) Western blotting detection of CLDN1 protein expression in XF04, and (**F**) relative expression of CLDN1 protein in XF04. Red, M60 control group; blue, XF04 experimental group. Data are expressed as ±standard deviation (SD). * *p* < 0.05, ** *p* < 0.001, *** *p* < 0.0001.

Based on the published literature and the patterns of differentially expressed mRNAs described above, we focused on 30 adhesion-promoting genes that were significantly downregulated in the suspension cells and 20 adhesion-repressing genes that were significantly upregulated in the suspension cells. To exclude possible false positives in the transcriptomics analysis, we performed a reverse transcription–quantitative polymerase chain reaction (RT-qPCR) validation of these 50 mRNAs. The primer sequences are listed in Table S1. Triplicate analyses showed that the expression patterns of the 33 mRNAs were consistent with the transcriptomics results. Among these 33 mRNAs, 13 were significantly upregulated in the suspension cells (Figure 3A,B), whereas 20 were significantly downregulated (Figure 3C,D). The RT-qPCR and Western blotting results (Figure 3B,E) showed that CLDN1 expression was significantly downregulated in the suspension MDCK cells, consistent with its reported role in promoting cell adhesion. Thus, we hypothesised that CLDN1 affects MDCK cell adhesion.

### 2.3. CLDN1 Promotes MDCK Cell Adhesion

**Figure 4:**
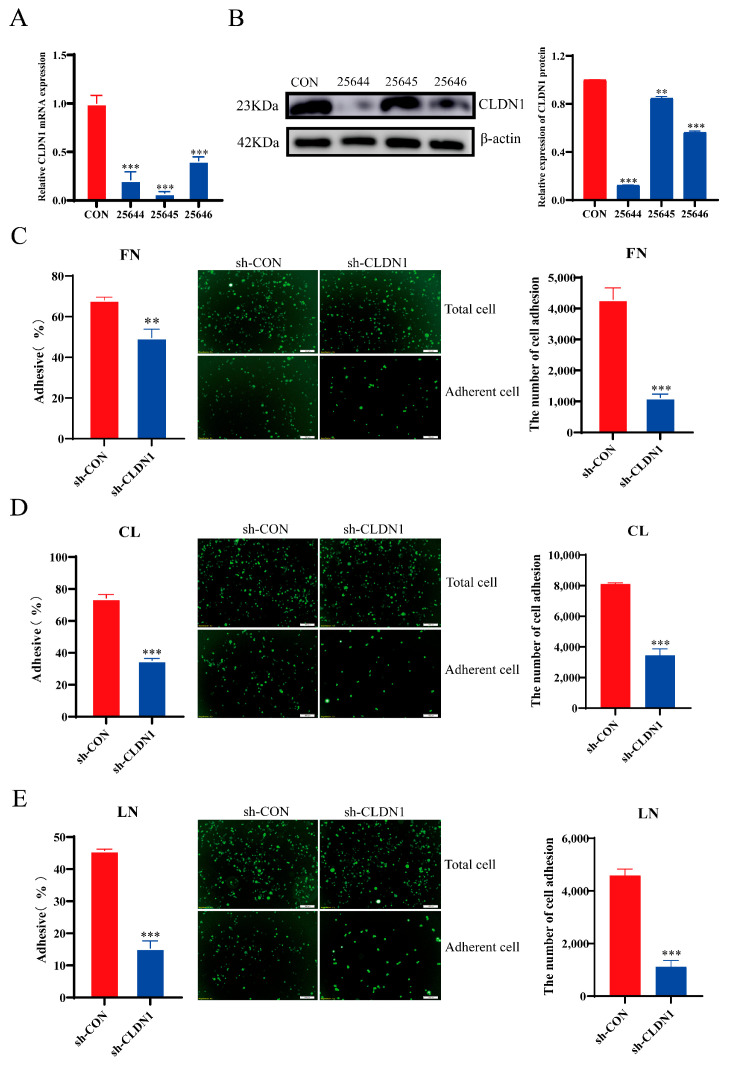
CLDN1 knockdown inhibits MDCK cell adhesion. (**A**) The RT-qPCR analysis to verify the expression of *CLDN1* mRNA in the sh-CLDN1 cells, and (**B**) the Western blotting analysis to verify the expression of CLDN1 protein in the sh-CLDN1 cells. (**C**) **Left**, CCK8 assay to detect the effect of CLDN1 knockdown on MDCK cell adhesion to the extracellular matrix protein fibronectin (FN); **middle**, fluorescence microscopy to detect the effect of CLDN1 knockdown on MDCK cell adhesion to FN; **right**, cell counting to detect the effect of CLDN1 knockdown on MDCK cell adhesion to FN. (**D**) **Left**, CCK8 assay to detect the effect of CLDN1 knockdown on MDCK cell adhesion to the extracellular matrix protein collagen I (CL); **middle**, fluorescence microscopy to detect the effect of CLDN1 knockdown on MDCK cell adhesion to CL; **right**, cell counting to detect the effect of CLDN1 knockdown on MDCK cell adhesion to CL. (**E**) **Left**, CCK8 assay to detect the effect of CLDN1 knockdown on MDCK cell adhesion to the extracellular matrix protein laminin (LN); **middle**, fluorescence microscopy to detect the effect of CLDN1 knockdown on MDCK cell adhesion to LN; **right**, cell counting to detect the effect of CLDN1 knockdown on MDCK cell adhesion to LN. Blue denotes CLDN1 knockdown cells (sh-CLDN1); red denotes empty vector control cells (sh-CON). Data are expressed as ±standard deviation. ** *p* < 0.01, *** *p* < 0.001.

**Figure 5:**
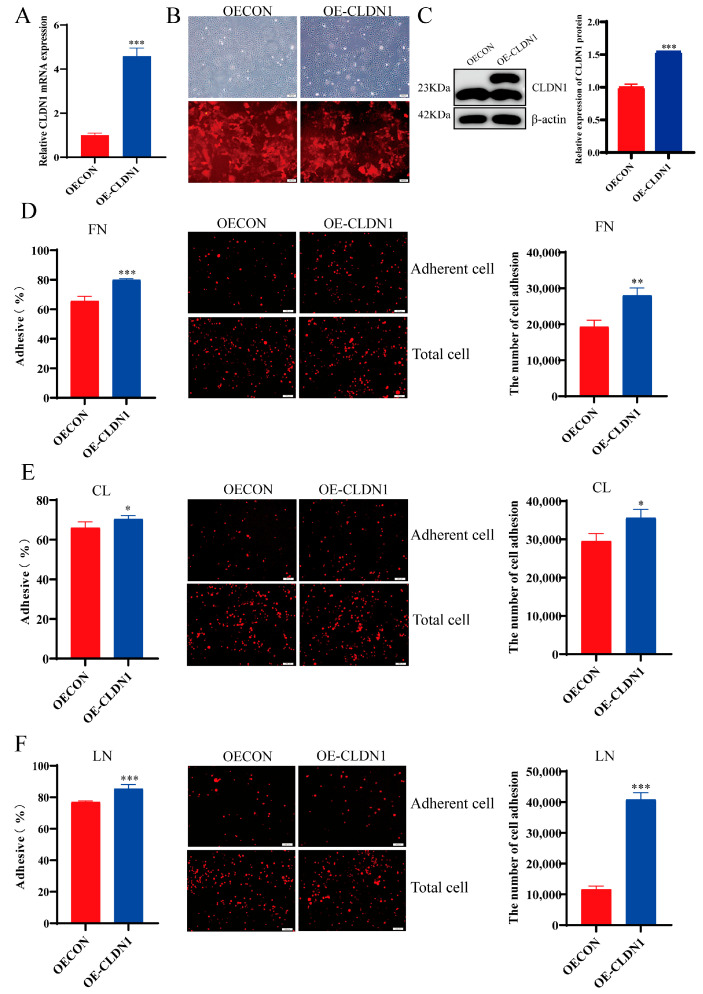
CLDN1 overexpression promotes MDCK cell adhesion. (**A**) The RT-qPCR analysis to verify the expression of *CLDN1* mRNA in the OE-CLDN1 cells, (**B**) lentiviral transfection for the fluorescence-based verification of expression, and (**C**) the Western blotting analysis to verify the expression of CLDN1 protein in the OE-CLDN1 cells. (**D**) **Left**, CCK8 assay to detect the effect of CLDN1 overexpression on MDCK cell adhesion to the extracellular matrix protein fibronectin (FN); **middle**, fluorescence microscopy to detect the effect of CLDN1 overexpression on MDCK cell adhesion to FN; **right**, cell counting to detect the effect of CLDN1 overexpression on MDCK cell adhesion to FN. (**E**) **Left**, CCK8 assay to detect the effect of CLDN1 overexpression on MDCK cell adhesion to the extracellular matrix protein collagen I (CL); **middle**, fluorescence microscopy assay to detect the effect of CLDN1 overexpression on MDCK cell adhesion to CL; **right**, cell counting to detect the effect of CLDN1 overexpression on MDCK cell adhesion to CL. (**F**) **Left**, CCK8 assay to detect the effect of CLDN1 overexpression on MDCK cell adhesion to the extracellular matrix protein laminin (LN); **middle**, fluorescence microscopy to detect the effect of CLDN1 overexpression on MDCK cell adhesion to LN; **right**, cell counting to detect the effect of CLDN1 overexpression on MDCK cell adhesion to LN. Blue denotes CLDN1 knockdown cells (OE-CLDN1); red denotes empty vector control cells (OE-CON). Data are expressed as ± standard deviation. * *p* < 0.05, ** *p* < 0.01, *** *p* < 0.001.

To investigate the effects of CLDN1 on MDCK cell adhesion, we constructed a *CLDN1* knockdown (sh-CLDN1) and overexpression cell lines (OE-CLDN1) in wild-type MDCK cells, using a lentiviral vector system. The RT-qPCR and Western blotting results showed that mRNA and protein levels of CLDN1 were significantly lower in the sh-CLDN1 cell lines than in the control group. The knockdown of the 25,644 target was the most significant, so this target-knocked-down cell was selected for subsequent experiments (Figure 4A,B). Furthermore, the CLDN1 mRNA and protein expression levels were significantly upregulated in the OE-CLDN1 cell line (Figure 5A,B). Next, we examined the effects of the CLDN1 knockdown and overexpression on MDCK cell adhesion using fluorescence detection, CCK8 assays, and cell counting. The results showed that the CLDN1 knockdown significantly suppressed MDCK cell adhesion to fibronectin (FN), collagen I (CL), and laminin (LN), compared with the control group (Figure 4C–E). In contrast, CLDN1 overexpression significantly promoted MDCK cell adhesion to FN, CL, and LN (Figure 5C–E). These findings collectively suggest that CLDN1 promotes MDCK cell adhesion.

### 2.4. CLDN1 Promotes MDCK Cell Proliferation and Migration

**Figure 6:**
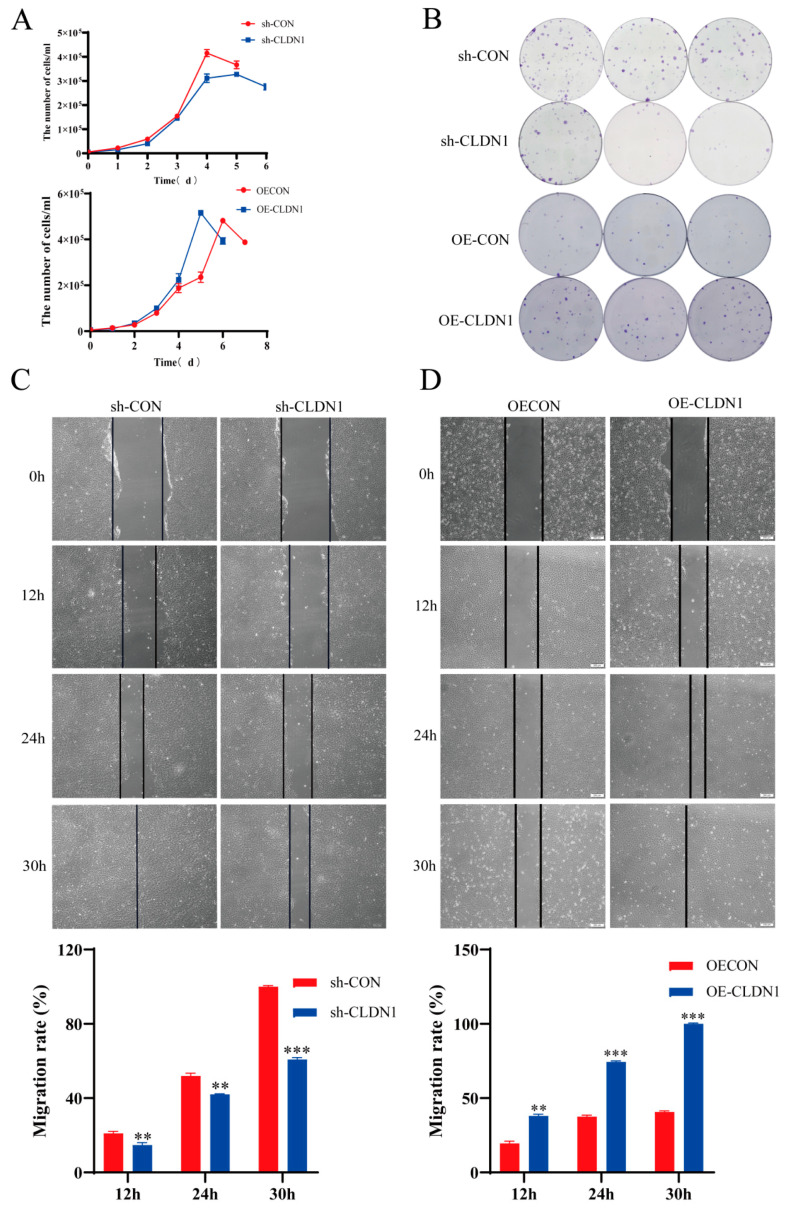
CLDN1 promotes MDCK cell proliferation and migration. (**A**) Cell counting was performed to determine the effects of the *CLDN1* knockdown and overexpression on MDCK cell proliferation. (**B**) The effects of *CLDN1* knockdown and overexpression on MDCK cell colony formation were determined by colony formation assays. (**C**,**D**) The effects of *CLDN1* knockdown and overexpression on MDCK cell migration were detected by scratch assays. ** *p* < 0.01, *** *p* < 0.001.

To investigate whether CLDN1 affects MDCK cell proliferation and migration, we analysed the proliferative and migratory capacities of the *CLDN1* knockdown and the overexpression cell lines. Cell growth curves and colony formation assays showed that the CLDN1 knockdown significantly suppressed MDCK cell proliferation, whereas *CLDN1* overexpression promoted MDCK cell proliferation (Figure 6A,B). Additionally, scratch assays showed that the *CLDN1* knockdown effectively inhibited MDCK cell migration (Figure 6C), whereas *CLDN1* overexpression enhanced MDCK cell migration (Figure 6D). These results collectively indicate that CLDN1 promotes MDCK cell proliferation and migration.

### 2.5. miR-175 Directly Targets the CLDN1 3′-UTR

**Figure 7:**
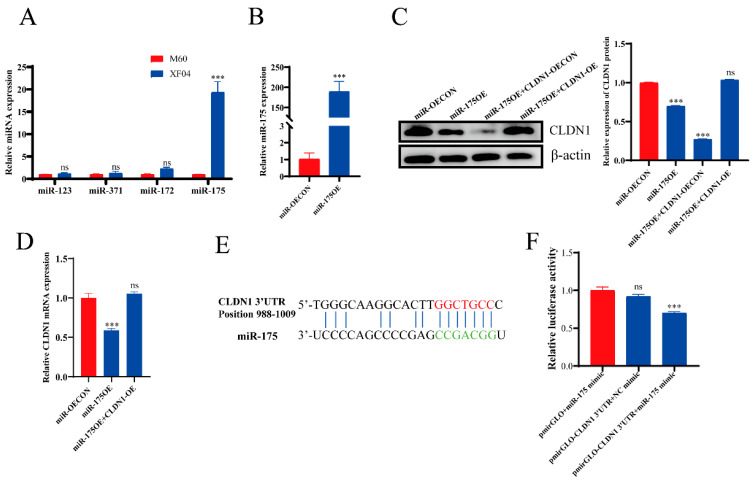
miR-175 directly targets the CLDN1 3′-UTR. (**A**) The RT-qPCR analysis to verify the expression of *CLDN1*-associated miRNAs. (**B**) The RT-qPCR analysis to verify the relative expression of miR-175. (**C**) The Western blotting analysis to detect miR-175 overexpression and relative protein expression of CLDN1 in functional rescue cells. (**D**) The RT-qPCR analysis to detect miR-175 overexpression and relative protein expression of CLDN1 in functionally rotating cells. (**E**) The schematic representation of the conserved miR-175 binding site within the *CLDN1* 3′-UTR and its potential interactions with the miR-175 sequence. (**F**) The dual luciferase reporter gene assay to detect the targeting relationship between miR-175 and *CLDN1*. miR-175 mimic or miR mimic control (mimic NC) were co-transfected with HEK293 cells for 48 h, and luciferase reporter gene activity was measured. All the measurements were performed in triplicate, and the experiment was repeated three times. Data are expressed as ±standard deviation. ns *p* > 0.05, *** *p* < 0.001.

Although multiple studies have shown that miRNAs play important roles in cell adhesion, none have identified miRNAs that regulate MDCK cell adhesion. Therefore, on the basis of clarifying that CLDN1 promotes the adhesion of MDCK cells, combined with the results of Small RNA-Seq analyses, we further predicted miRNAs matching *CLDN1*, and a total of miR-123, miR-172, miR-175, and miR-371 were predicted as possible binding to *CLDN1* 3′-UTR targets. miR-172, miR-175, and miR-371 may bind to a *CLDN1* 3′-UTR target. The RT-qPCR verification of the expression levels of these miRNAs showed that miR-175 expression was significantly upregulated in the suspension MDCK cells compared with the adherent MDCK cells (Figure 7A). These findings were consistent with the transcriptomics and inversely related to the expression levels of the target genes, suggesting that miR-175 is involved in MDCK cell adhesion. To confirm that miR-175 targets *CLDN1*, we constructed miR-175 overexpression cell lines using a lentiviral vector system. The RT-qPCR analysis confirmed the successful construction of miR-175 overexpression cell lines (Figure 7B). It also showed that miR-175 overexpression significantly suppressed CLDN1 expression at both the mRNA and protein levels, whereas *CLDN1* overexpression in the context of miR-175 overexpression was able to restore *CLDN1* expression to wild-type levels (Figure 7C,D).

To elucidate the mechanism by which miR-175 regulates CLDN1 expression, we investigated interactions between miR-175 and the *CLDN1* mRNA 3′-UTR. Sequence comparison using DNA-MAN software (v10) revealed the presence of an miR-175 binding site within the *CLDN1* 3′-UTR (Figure 7E). To demonstrate that miR-175 binds to the *CLDN1* 3′-UTR, thus regulating the expression of CLDN1, we performed a dual-luciferase reporter gene assay that involved co-transfecting HEK293 cells with an miR-175 mimic and a dual-luciferase plasmid vector (pmiRGLO-CLDN1) containing the miR-175 binding site within the *CLDN1* 3′-UTR. The analysis of the dual-luciferase reporter gene activity showed that the co-transfection of the *CLDN1* 3′-UTR and an miR-mimic NC did not influence dual-luciferase activity, whereas the co-transfection of *CLDN1* 3′-UTR and the miR-175 mimic significantly suppressed dual-luciferase activity (Figure 7F). Collectively, these results suggest that miR-175 inhibits *CLDN1* expression by targeting the *CLDN1* 3′-UTR.

### 2.6. miR-175 Inhibits MDCK Cell Adhesion by Targeting CLDN1

**Figure 8:**
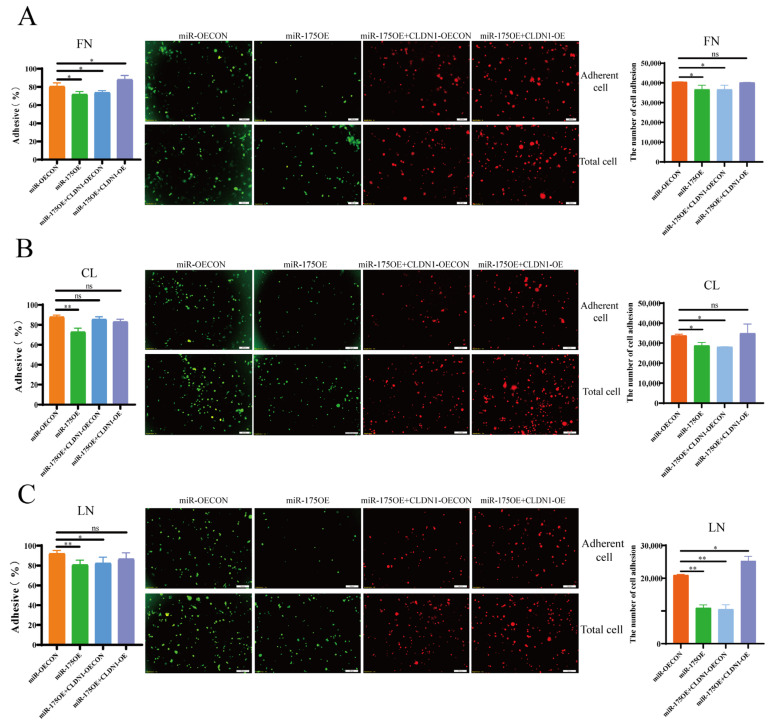
miR-175 inhibits MDCK cell adhesion by targeting *CLDN1*. (**A**) **Left**, 96-well plates were coated with fibronectin (FN) and the effects of miR-175OE or miR-175OE + CLDN1-OE on MDCK cell adhesion were detected by CCK8 assays; centre, 24-well plates were coated with FN and adherent cells were detected by fluorescence microscopy; **right**, adherent cells were counted in 24-well plates coated with FN. (**B**) **Left**, 96-well plates were coated with collagen I (CL) and the effects of miR-175OE or miR-175OE + CLDN1-OE on MDCK cell adhesion were detected by CCK8 assays; centre, 24-well plates were coated with CL and adherent cells were detected by fluorescence microscopy; **right**, 24-well plates were coated with CL and adherent cells were counted. (**C**) **Left**, 96-well plates were coated with laminin (LN) and the effects of miR-175OE or miR-175OE + CLDN1-OE on MDCK cell adhesion were detected by CCK8 assays; centre, 24-well plates were coated with LN and adherent cells were detected by fluorescence microscopy; **right**, 24-well plates were coated with LN and adherent cells were counted. Orange, miR-175 overexpression control cells (miR-OECON); green, miR-175 overexpression cells (miR-175OE); blue, target gene CLDN1 rescue control cells (miR-175OE + CLDN1-OECON); purple, target gene CLDN1 rescue cells (miR-175OE + CLDN1-OE). Data are expressed as ± standard deviation. ns *p* > 0.05, * *p* < 0.05, ** *p* < 0.01.

Our results thus far have demonstrated that miR-175 inhibits CLDN1 expression via binding to the *CLDN1* 3′-UTR. Next, we investigated the effect of miR-175 binding to *CLDN1* on MDCK cell adhesion using CCK8, fluorescence detection, and cell counting assays. The results showed that miR-175 overexpression inhibited MDCK cell adhesion to the extracellular matrix proteins FN, CL, and LN (Figure 8A–C), suggesting that miR-175 inhibited MDCK cell adhesion. Then, the overexpression of *CLDN1* on the basis of miR-175 overexpression inhibited the inhibitory effect of miR-175 on MDCK cell adhesion, suggesting that *CLDN1* overexpression can rescue the effect of miR-175 overexpression on MDCK cell adhesion (Figure 8A–C). Overall, the above results suggest that miR-175 inhibits MDCK cell adhesion by targeting the *CLDN1* 3′-UTR.

### 2.7. miR-175 Targets CLDN1 to Inhibit MDCK Cell Proliferation and Migration

**Figure 9:**
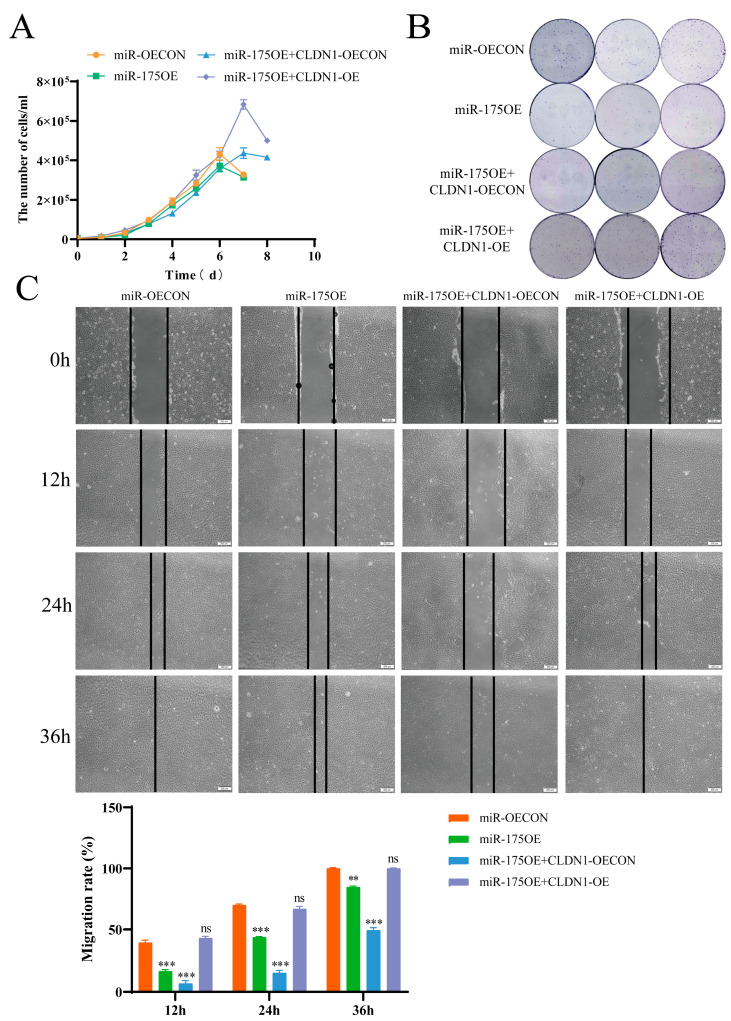
miR-175 inhibits MDCK cell proliferation and migration. (**A**) The effects of miR-175OE or miR-175OE + CLDN1-OE on MDCK cell proliferation were determined by cell counting. (**B**) The effects of miR-175OE or miR-175OE + CLDN1-OE on MDCK cell colony formation were determined by colony formation assays. (**C**) The effects of miR-175OE or miR-175OE + CLDN1-OE on MDCK cell migration were determined by cell scratch. Orange, miR-175 overexpression control cells (miR-OECON); green, miR-175 overexpression cells (miR-175OE); blue, target gene CLDN1 rescue control cells (miR-175OE + CLDN1-OECON); purple, target gene *CLDN1* rescue cells (miR-175OE + CLDN1-OE). Data are expressed as ± standard deviation. ns *p* > 0.05, ** *p* < 0.01, *** *p* < 0.001.

Considering that CLDN1 promotes MDCK cell proliferation and migration, we explored whether miR-175 affects MDCK cell proliferation and migration. Cell growth curves and colony formation assays preliminarily showed that miR-175 overexpression inhibited MDCK cell proliferation, whereas simultaneous *CLDN1* overexpression reversed the inhibitory effect of miR-175 on MDCK cell proliferation (Figure 9A,B). Additionally, cell scratch assays showed that miR-175 overexpression inhibited MDCK cell migration, whereas *CLDN1* overexpression reversed the inhibitory effect of miR-175 on MDCK cell migration (Figure 9C). The above results suggest that miR-175 inhibits MDCK cell proliferation and migration by targeting *CLDN1*.

## 3. Discussion

MDCK cells, the preferred cell line for influenza virus proliferation and vaccine production, are epithelial cells that typically grow in an adherent manner and require surface adhesion for proliferation; this requirement limits the expansion of the influenza virus and the production of vaccines. The suspension culturing of adherent cells via transfection or domestication techniques would be useful for vaccine production and a key factor is the mechanism underlying cell adhesion. Suspension cells can be freed from the limitation of the surface area of the culture vector, realizing a high-density culture and significantly increasing the vaccine yield. These cell lines can be obtained by a variety of methods, such as suspension domestication and genetic engineering techniques. It has been reported that a variety of suspension cells, including MDCK [23], such as Chinese hamster ovary (CHO) [24], Vero [25], human embryonic kidney (HEK) 293 [26], baby hamster kidney-21 (BHK-21) [27], etc., have been obtained by suspension domestication. Although many suspension cell lines have been obtained by suspension domestication techniques, these methods still have some drawbacks, such as a long suspension domestication cycle, a high number of cell passages, and the potential risk of reduced cell proliferative activity and viral susceptibility [28]. Gene editing technology has received increasing attention as a new method to alter cellular traits by modifying the expression of functional genes in cells. And the key to constructing suspension-adapted cell lines by genetic engineering modification is to understand cell adhesion-related genes.

The cell adhesion process is regulated by many adhesion molecules and transmembrane adhesion proteins that mediate cell–cell and cell–extracellular matrix interactions [29]. However, there remains a lack of clarity concerning the mechanisms of MDCK cell adhesion. In this study, we used RNA-Seq transcriptomics to identify differentially expressed mRNAs and miRNAs between adherent and suspension MDCK cells. In total, we detected 50,603 differentially expressed mRNAs and 744 differentially expressed miRNAs. Combined GO and KEGG analyses revealed 743 differentially expressed mRNAs involved in cell adhesion. Further GO and KEGG analyses of these mRNAs showed that they were mainly involved in the cell adhesion process and primarily localised at the plasma membrane; their molecular functions were predominantly related to calmodulin binding.

Our results showed that miR-175, miR-60, miR-116, and miR-415 were significantly upregulated in the suspension MDCK cells compared with the adherent MDCK cells. According to multiple databases, miR-175 may bind to mRNAs such as *CLDN1*, *CLDN4*, and *TLN2*; however, there is a lack of clarity regarding the biological functions of miR-175. In the present study, we confirmed that miR-175 inhibited MDCK cell adhesion to the extracellular matrix proteins FN, CL, and LN via binding to the *CLDN1* 3′-UTR. Further investigation is needed to determine whether miR-175 also regulates MDCK cell adhesion by targeting *CLDN4* and *TLN2*.

CLDNs constitute a family of membrane proteins, mainly present in endothelial or epithelial cells, that are essential for cell adhesion and migration [30]. As a tight junction protein, CLDN1 is an integral membrane protein and a component of the tight junction [31]. Several studies have shown that CLDN1 can interact with E-cadherin and β-catenin to regulate diverse intercellular adhesion processes [18,32]. However, no studies have demonstrated whether CLDN1 regulates cell–extracellular matrix adhesion. In the CLDN family, CLDN5 expression is regulated by integrin β1-mediated brain endothelial cell–extracellular matrix adhesion, which affects brain microvessel permeability [33]. Furthermore, CLDN7 forms a protein complex with integrin β1 in human lung cancer cells; CLDN7 overexpression leads to greater surface adhesion in cell culture and higher expression levels of adhesion proteins, indicating that CLDN7 promotes cell–extracellular matrix adhesion via integrin β1 [34]. Accordingly, we hypothesised that CLDN1 also affects cell–extracellular matrix adhesion; to our knowledge, no studies have explored the effects of CLDN1 on MDCK cell adhesion. In the present study, we showed that CLDN1 promotes MDCK cell adhesion to the extracellular matrix proteins FN, CL, and LN. Therefore, we speculate that CLDN1 can serve as a target for genetic engineering to create the suspension MDCK cells.

Because cell adhesion is regulated by multiple adhesion factors, the acquisition of suspension cells may require multi-gene editing. The differentially expressed genes identified in this study—for example, desmoglein 3 (DSG3) and tight junction protein 2 (TJN2), which reportedly regulate cell–cell adhesion [35,36]; the extracellular matrix proteins cellular communication network factor 1 (CCN1) and netrin 1 (NTN1); and the transmembrane proteins dipeptidyl peptidase 4 (DPP4) and integrin subunit α 6 (ITGA6), which mainly regulate cell–extracellular matrix adhesion [37,38,39,40]—may also be important genes affecting MDCK cell adhesion. These genes can serve as candidate targets for the establishment of genetically engineered suspension MDCK cells via multi-gene editing. Therefore, we hypothesise that simultaneous miR-175 overexpression and CLDN1 knockdown can be used to establish suspension-adapted MDCK cells, offering a new approach for generating the suspension MDCK cells via genetic engineering and facilitating vaccine production.

Disrupted cell adhesion and changes in adhesion protein expression patterns are hallmarks of cancer invasion and metastasis. CLDN1 has been implicated in various cancers; changes in its expression levels have substantial effects on cell migration, invasion, and proliferation. Reduced CLDN1 expression has been positively associated with poor prognoses in colon cancer [41], as well as tumour recurrence in lung adenocarcinoma [42] and breast cancer [43]. Conversely, increased CLDN1 expression has been associated with enhanced invasive and metastatic behaviours in colon, hepatocellular, and oral cancers [44,45,46]. Overall, CLDN1 has been associated with diseases such as tumour development and epithelial dysfunction; we speculate that CLDN1 is linked to the prognosis of various other diseases.

There is evidence that low CLDN1 expression is associated with a poor prognosis in patients with triple-negative breast cancer. Additionally, CLDN1 overexpression increases the sensitivity of triple-negative breast cancer cell lines to the chemotherapeutic agents 5-fluorouracil, paclitaxel, and doxorubicin [47]. Cherradi et al. found that CLDN1 expression was upregulated in oxaliplatin-resistant colorectal cancer, and CLDN1 knockdown promoted the sensitivity of colorectal cancer cells to oxaliplatin [48]. These findings indicate that CLDN1 can serve as a novel biomarker of chemotherapy-acquired resistance in colorectal cancer patients, helping to avoid the development of resistance and improving the prognoses of advanced colorectal cancer patients. In non-small-cell lung cancer, CLDN1 is involved in the development of chemotherapeutic resistance to anticancer drugs such as cisplatin, doxorubicin, SN-38, and gemcitabine [49]. Furthermore, CLDN1 activates autophagy and promotes resistance to cisplatin in non-small-cell lung cancer cells by upregulating Unc-51-like kinase 1 (ULK1) phosphorylation [50].

Although CLDN1 has been examined in numerous cell types, its precise biological role in MDCK cells has remained unclear. In the present study, we preliminarily explored the effects of CLDN1 on MDCK cell proliferation and migration. We found that CLDN1 promoted MDCK cell proliferation and migration, whereas miR-175 inhibited MDCK cell proliferation and migration, which was opposite to the function of its target gene *CLDN1*. Therefore, we speculate that CLDN1 is associated with MDCK cell proliferation and migration. Our findings offer a potential target for the treatment of renal epithelial-related diseases. Nevertheless, the specific mechanism by which CLDN1 regulates MDCK cell proliferation and migration requires further exploration.

## 4. Materials and Methods

### 4.1. Cell Culture

HEK293: purchased from the American Type Culture Collection (ATCC). Adherent culture type MDCK cells: introduced from the ATCC; after introduction, the main cell bank (MDCK-M60, P60) was established by the Gansu Animal Cell Technology Innovation Centre in accordance with the requirements of the Chinese Pharmacopoeia (three parts) 2010 edition, and the cells were cultured with a DMEM medium (Lanzhou Bailing Biotechnology Co., Ltd., Lanzhou, China, BGL M101.01) containing 10% Newborn bovine serum (NBS, CellMax, Beijing, China, SA311.01) in a DMEM medium. Suspension-cultured MDCK cells: XF04 was previously obtained in our laboratory by suspension domestication and cultured with a serum-free medium (SFM, CellMax, CFM414.08) in shake flasks (431401, Corning, London, UK), 5% CO_2_, 37 °C, 120 r/min [51].

### 4.2. mRNA Library Establishment

The library preparation was performed using an Optimal Dual-mode mRNA Library Prep Kit (BGI Geneomics, Shenzhen, China). A certain amount of RNA was denatured at a suitable temperature to open the secondary structure, and the mRNA was enriched by oligo (dT)-attached magnetic beads. After reacting at a suitable temperature for a fixed time period, the RNA was fragmented with fragmentation reagents. Then, first-strand cDNA was generated using random, hexamer-primed reverse transcription, followed by a second-strand cDNA synthesis. The synthesized double strand cDNA was subject to an end repairment reaction. After the cDNA end repairment, a single ‘A’ nucleotide was added to the 3′ ends of the blunt fragments through an A-tailing reaction. Then, the reaction system for adaptor ligation configured to ligate adaptors with the cDNA, and finally, the library products were amplified through a PCR reaction and subjected to quality control. Next, the single-stranded library products were produced via denaturation. The reaction system for circularization was set up to get the single-stranded cyclized DNA products. Any uncyclized, single-stranded linear DNA molecules were digested. The final single-stranded, circularized library was amplified with phi29 and rolling circle amplification (RCA) to make a DNA nanoball (DNB), which carried more than 300 copies of the initial single-stranded, circularized library molecule. The DNBs were loaded into the patterned nanoarray and PE 100/150 bases reads were generated on a G400/T7/T10 platform (BGI Geneomics, Shenzhen, China).

### 4.3. miRNA Library Establishment

The library preparation was performed using an MGIEasy Small RNA Library Prep Kit (BGI Geneomics, Shenzhen, China). A certain amount of RNA was ligated by 3′ and 5′ adaptors sequentially in two reaction systems for adaptor ligation. After the adaptor ligation, the RNA was reverse transcribed into cDNA and was PCR amplified. The library was fragment-size-selected by polyacrylamide gel electrophoresis and subjected to quality control. Next, the single-stranded library products were produced via denaturation. The reaction system for circularization was set up to get the single-stranded, circularized DNA products. Any single-stranded linear DNA molecules were digested. The final single-stranded, circularized library was amplified with phi29 and rolling circle amplification (RCA) to make a DNA nanoball (DNB), which carried more than 300 copies of the initial single-stranded, circularized library molecule. The DNBs were loaded into the patterned nanoarray and sequencing reads of SE50 bases length were generated on a G400 platform (BGI Geneomics, Shenzhen, China).

### 4.4. Reverse Transcription and Polymerase Chain Reaction

The total RNA from the MDCK cell lines was prepared by a Trizol reagent (Accurate Biotech, Changsha, China), according to the manufacturer’s protocol, and the concentrations were determined using a NanoDrop 2000 spectrophotometer (Thermo Fisher Scientific, Waltham, MA, USA). For miRNA quantification, cDNA was synthesised using the miRNA 1st Strand cDNA Synthesis Kit (by tailing A) (Vazyme Biotech, Nanjing, China), and real-time fluorescence was performed using the miRNA Universal SYBR qPCR Master Mix (Vazyme Biotech) quantitative PCR, using *U6* as an internal reference gene for the miRNA. Reaction conditions: denaturation at 95 °C for 5 min; annealing at 95 °C for 10 s and 60 °C for 30 s for a total of 40 cycles; extension at 95 °C for 15 s, 60 °C for 60 s, and 95 °C for 15 s for a total of one cycle. For mRNA quantification, cDNA was synthesised using the Evo M-MLV Reverse Transcription Kit (Accurate Biotech), and real-time fluorescent quantitative PCR was performed using the SYBR Green Pro Taq HS Premixed qPCR Kit (Accurate Biotech) and gene-specific primers, with *GAPDH* as the internal reference gene. Reaction conditions: pre-denaturation at 95 °C for 30 s, denaturation at 95 °C for 5 s, annealing at 60 °C for 30 s, and a total of 40 cycles. Using the 2^−ΔΔCt^ method (DOI: 10.1006/meth.2001.1262), this was performed on a real-time PCR device (Bio-RAD, California, USA, CFX96). The primer sequences involved in this study are shown in Table S1. All the primers were designed by the Hunan Accurate Biological Engineering Co. (Changsha, China).

### 4.5. Western Bloting

MDCK cells were collected and total protein was prepared by adding RIPA lysate (EpiZyme, Shanghai, China), lysed on ice for 30 min, and centrifuged at 4 °C and 12,000 rpm for 10 min, and then the supernatant was taken and the protein concentration was determined by the BCA method (EpiZyme). After SDS-PAGE gel electrophoresis, the proteins were transferred to a PVDF membrane, which was closed with TBST solution containing 5% skimmed milk (Solaibio, Beijing, China, LP0033B) for 2 h at room temperature; the primary antibody was added and placed for overnight incubation at 4 °C, and the membrane was washed by TBST 3 times, each time for 5 min; the corresponding secondary antibody was diluted with the closure solution, and incubated for 1 h at room temperature, and the membrane was washed by TBST for 3 times, each time for 5 min; ECL chemiluminescent solution (meilunbio) was added dropwise, and a chemiluminescent instrument (Beijing Sage Venture Technology Co., Ltd. Beijing, China) was set up to measure protein levels. The primary antibodies involved in this assay included CLDN1 (Proteintech, Wuhai, China, Cat No.13050-1-AP, dilution ratio: 1:1000), β-actin (Proteintech, Cat No.20536-1-AP, dilution ratio: 1:2000), and GAPDH (Proteintech, Cat No.10494-1-AP, dilution ratio: 1:5000), and the secondary antibody was rabbit antibody (Proteintech, Cat No.SA00001-2, dilution ratio: 1:5000).

### 4.6. miR-175 Overexpression, CLDN1 Knockdown, Overexpression Cell Construction, and the Screening of Stable Cell Lines

MDCK cells were homogeneously inoculated into 24-well plates. miR-175OE plasmid, sh-CLDN1 plasmid, CLDN1-OE plasmid, and negative control plasmid were transfected into the cells by lentivirus (OBiO, Shanghai, China) the next day, when the cell density reached 60%–70%. After 12 h, the medium was replaced with DMEM, containing 10% fetal bovine serum, and the cells were incubated for 48 h in a 5% CO_2,_ 37 °C incubator, fluorescence expression was observed under a fluorescence microscope, and the cells were passed on for culture. After the cells were attached to the wall, puromycin at a concentration of 4 μg/mL (Solarbio) or 12 μg/mL blasticidin (Solarbio)was added to screen for resistant cells. Stable cell lines with miR-175 overexpression and *CLDN1* knockdown and overexpression were obtained after 15 consecutive generations of screening.

### 4.7. Dual Luciferase Reporter Gene Test

The reporter plasmid carrying the wild-type 3′UTR was constructed as follows. The 3′ UTR of *CLDN1* (ID: 608207 position 635–1059 of CLDN1 3′ UTR) was amplified from MDCK genomic DNA using the following primers: PmeI-CLDN1-3′ UTR forward primer 5′-GGGGTTTAAACTCTGGTAGAGAGGCGGTGTGAG-3′ and SalI-CLDN1-3′ UTR reverse primer 5′-GCGTCGACGGCAGGTCCAGAATTACAAT-3′. The 3′ UTR wild-type sequence was enzymatically cut by PmeI (Beyotime, Shanghai, China, D6542S) and SalI (Beyotime, D6598S) to the 3′UTR wild-type. The sequence was cloned into the pmiRGLO dual luciferase miRNA target expression vector (Promega, Madison, WI, USA) and the insertion sequence was confirmed by DNA sequencing. Reaction conditions: pre-denaturation at 95 °C for 3 min, denaturation at 95 °C for 15 s, annealing at 56 °C for 15 s, and extension at 72 °C for 45 s, a total of 30 cycles, and finally thorough extension at 72 °C for 5 min. This was performed on a PCR device (CLASSIC, K960, Hangzhou Jingge Scientific Instrument Co. Hangzhou, China). Ampicillin (AMP) was used for colony screening. HEK293 cells were transiently transfected with miR-175 mimic (UGGCAGCCGAGCCCCGACCCCU), miR mimic control (CON), and pmiRGLO-3′UTR according to the transfection instructions of the lipofectamine 3000 reagent (Hanhen Bio, Shanghai, China), and 48 h after transfection, a luciferase assay and luminescence was measured using a GloMax EXPLORER device (Promega, Madison, WI, USA) using the default parameters. miR-175 mimic and miR mimic NC (catalog numbers:miR1N0000001-1-5) were synthesised by RioBio (Shenzhen, China).

### 4.8. Cell Proliferation Experiments

An MDCK cell suspension with a good growth condition was prepared by trypsin digestion, and the cell concentration was adjusted to 5 × 10^3^ cells/mL, inoculated in 24-well cell culture plates, 1 mL per well, and cultured in an incubator with 5% CO_2_ at 37 °C. The cells were prepared as a cell suspension by digesting them with trypsin every 24 h, and a small amount of the suspension was added to a cell counting plate and placed in a cell counter (Countstar, Alit, Shanghai, China) for counting. Three replicates were performed in parallel in each group for 10 days. The mean values were calculated and cell growth curves were plotted.

### 4.9. Cell Migration Assay

MDCK cells in the logarithmic growth phase were taken and digested and counted by trypsin, and then the cells were inoculated into six wells with 6 × 10^5^ cells per well; after the cells were fully grown, the central area of the cell monolayer was scratched along a straight line with a 200 μL pipette tip, the scratched cells were washed with PBS, serum-free DMEM was added, and the width of the scratches was photographed with an inverted microscope at 0 h. The cells were incubated in a constant temperature incubator at 5% CO_2_, 37 °C for 6, 12, 24, and 36 h. After washing with PBS, the cells were observed under an inverted microscope (OLYMPUS, CKX41SF), photographed at the same position, and measured with Image J (v1.53e) (https://imagej.net/software/imagej/ (accessed on 1 December 2023) software.

### 4.10. Cell Adhesion Assay

CCK8 assay for adhesion rate: Using 10 μg/mL fibronectin (FN, Solarbio, Beijing, China, F8180), collagen I (CL, Solarbio, C8061), laminin (LN, Solarbio, CLP0114) in 96-well plates were incubated at 37 °C for 12 h, washed three times with PBS, and then closed with serum-free DMEM containing 1% BSA (Solarbio, SA8130) for 1 h. In each well, 1 × 10^4^ cells were incubated at 37 °C for 30 min, and non-adherent and loosely adherent cells were washed with PBS. At 30 min, cells with no wall adherence and loose wall adherence were washed with PBS, the remaining adherent cells were added to CCK8 (Solarbio, CA1210) and incubated for 1 h, and then the absorbance value at 450 nm was measured; the cell adhesion rate = (OD value of the experimental group—OD value of the blank control)/(OD value of the control group OD value of the blank control). Adherent cell count: 5 × 10^4^ cells were added to the 24-well plate and incubated at 37 °C for 30 min; after the cells with no adherence and loose adherence were washed with PBS, the adherent cells were counted and the field of view was randomly selected for fluorescence detection under the microscope (OLYMPUS, Tokyo, Japan, U-TV0.63XC).

### 4.11. mRNA and miRNA Bioinformatics Analysis

Data filtering: The raw data were filtered with SOAPnuke (v1.5.0) [52] by (1) removing reads containing adapters (adapter contamination); (2) removing reads whose unknown base (‘N’ base) ratio was more than 1%; (3) removing reads whose low-quality base ratio (base quality less than or equal to 15) was more than 40%. Afterwards, clean reads were obtained and stored in an FASTQ format. Reference genome mapping: the clean data were mapped to the reference genome by HISAT (v2.2.1) [53]. Reference gene mapping: the clean data were mapped to the assembled unique gene by Bowtie2 (v2.4.5) [54]. The expression level of genes was calculated by RSEM (v1.3.1) [55]. Gene Annotation: we used the public database Gene Ontology (GO) [56] and the Kyoto Encyclopedia of Genes and Genomes (KEGG) [57] to annotate the genes. Time Series and WGCNA analyses: a time series analysis was performed by Mfuzz (v2.60.0) [58], and a gene co-expression network analysis was performed by WGCNA (v1.71). Differential gene expression: a between-group differential gene analysis was performed using DEGseq [59] under the conditions of Fold Change ≥ 2 and Adjusted *p* value ≤ 0.001. PoissonDis was performed following the between-sample differential gene analysis under the conditions of Fold Change ≥ 2 and FDR ≤ 0.001. Using the pheatmap function on the differential gene, set to draw a heatmap of differential gene clusters. According to the GO and KEGG annotation results and classifications, the differentially expressed genes were functionally classified, the phyper in R software was used for the KEGG enrichment analysis, and the TermFinder package was used for the GO Enrichment analysis. With a Q value of ≤0.05 as the threshold, candidate genes that met this condition were defined as significantly enriched.

Small RNA annotation and miRNA identification: The raw sequencing data were called raw tags. We obtained clean tags using the SOAPnuke [52]. The filtering rules were as follows: remove low quality tags (the number of bases with base quality less than 10 is ≥4, or the number of bases with base quality less than 13 is ≥6); remove tags with 5′ adaptor contaminants; remove tags without 3′ adaptor; remove tags without insertion; remove tags with poly A; remove tags shorter than 18nt. After filtering, the clean tags were mapped to the reference genome, miRbase (V22), and other sRNA databases with Bowtie2 [60]. Particularly, a cmsearch [61] was performed for Rfam (V13.0) mapping. Annotation information, as well as the alignment of sRNA to different RNAs, were summarized. Multiple categories annotated for a sRNA appeared. To ensure that each small RNA was mapped to a unique category, we set the following priority rule: MiRbase > pirnabank > snoRNA > Rfam > other sRNA. The MiRDeep2 (https://github.com/rajewsky-lab/mirdeep2 (accessed on 1 February 2022) package was used to identify potential novel miRNAs.

The differential expression analysis of miRNA and the target gene analysis: The small RNA expression level was calculated by counting the absolute numbers of molecules using unique molecular identifiers [62]. The expression formula was Expression = C × 1,000,000/T, C was the number of UMIs of reads that were mapped to the small RNA, T was the UMI number of total clean reads of the sample. The differential expression analysis was performed using the DEGseq [59], Q value ≤ 0.001 (or Q ≤ 0.05) and the absolute value of Log_2_FoldChange ≥ 1 (or 0) as the default threshold to judge the significance of expression difference. RNAhybrid [63], miRanda [64], and TargetScan [65] were used to predict the target genes of miRNAs. To annotate the gene functions, all the target genes were aligned against KEGG [57] (v89.1) and GO [56]. The GO enrichment analysis and the KEGG enrichment analysis of target genes were performed using phyper, a function of R (R-3.1.1). The *p*-value was corrected using the Bonferroni method [66], and a corrected *p*-value ≤ 0.05 was taken as a threshold. The GO terms or KEGG terms fulfilling this condition were defined as significantly enriched terms.

All the valid URLs of the bioinformatics analysis software used in this experiment are shown in Table S2.

### 4.12. Statistical Analysis

The experimental data are expressed as “±standard deviation”, and Graphpad Prism 9.0 (https://www.graphpad.com/scientific-software/prism/ (accessed on 15 December 2023) was used for the one-way analysis of variance (ANOVA). * *p* < 0.05, ** *p* < 0.001, *** *p* < 0.0001.

## Data Availability

All the data generated or analysed during this study are included in this article and its additional files. All the RNA-seq data analysed during this study have been deposited in the SRA database (https://www.ncbi.nlm.nih.gov/sra/PRJN (accessed on 30 September 2024)) under the accession number SUB14758911.

## Supplementary Materials

The following supporting information can be downloaded at https://www.mdpi.com/article/10.3390/genes15101333/s1, Table S1: Primer sequence information, Table S2: All the valid URLs of the bioinformatics analysis software.

## References

[1] Ren Z.H., Lu Z.Z., Wang L. (2015). Rapid production of a H9N2 influenza vaccine from MDCK cells for protecting chicken against influenza virus infection. Microbiol. Biotechnol..

[2] Itsuki H., Hitoshi T., Shimazaki N. (2022). Suitability of NIID-MDCK cells as a substrate for cell-based influenza vaccine development from the perspective of adventitious virus susceptibility. Microbiol. Immunol..

[3] Alexander D., Scott A. (2009). Trivalent MDCK cell culture-derived influenza vaccine Optaflu^®^ (Novartis Vaccines). Expert Rev. Vaccines.

[4] Pech S., Rehberg M., Janke R., Benndorf D., Genzel Y., Muth T., Sickmann A., Rapp E. (2021). Tracking changes in adaptation to suspension growth for MDCK cells: Cell growth correlates with levels of metabolites, enzymes and proteins. Microbiol. Biotechnol..

[5] Chu C., Lugovtsev V., Golding H., Betenbaugh M., Shiloach J. (2009). Conversion of MDCK cell line to suspension culture by transfecting with human siat7e gene and its application for influenza virus production. Proc. Natl. Acad. Sci. USA.

[6] Gregory P.A., Bert A.G., Paterson E.L., Barry S.C., Tsykin A., Farshid G., Vadas M.A., Khew-Goodall Y., Goodall G.J. (2008). The miR-200 family and miR-205 regulate epithelial to mesenchymal transition by targeting ZEB1 and SIP1. Nat. Cell Biol..

[7] Obeng G., Park E.J., Appiah M.G., Kawamoto E., Gaowa A., Shimaoka M. (2021). miRNA-200c-3p targets talin-1 to regulate integrin-mediated cell adhesion. Sci. Rep..

[8] Hunt S., Jones A.V., Hinsley E.E., Whawell S.A., Lambert D.W. (2010). MicroRNA-124 suppresses oral squamous cell carcinoma motility by targeting ITGB1. FEBS Lett..

[9] Hozaka Y., Seki N., Tanaka T., Asai S., Moriya S., Idichi T., Wada M., Tanoue K., Kawasaki Y., Mataki Y. (2021). Molecular Pathogenesis and Regulation of the miR-29-3p-Family: Involvement of ITGA6 and ITGB1 in Intra-Hepatic Cholangiocarcinoma. Cancers.

[10] Gong C., Yang Z., Wu F., Han L., Liu Y., Gong W. (2016). miR-17 inhibits ovarian cancer cell peritoneal metastasis by targeting ITGA5 and ITGB1. Oncol. Rep..

[11] Carolina O.R., María C.O., Rafael S.F. (2022). Hsa-miR-183-5p Modulates Cell Adhesion by Repression of ITGB1 Expression in Prostate Cancer. Non-Coding RNA.

[12] Qin Q., Wei F., Zhang J., Li B. (2017). miR-134 suppresses the migration and invasion of non-small cell lung cancer by targeting ITGB1. Oncol. Rep..

[13] Zhu X., Jin X., Li Z., Chen X., Zhao J. (2023). miR-152-3p facilitates cell adhesion and hepatic metastases in colorectal cancer via targeting AQP11. Pathol. Res. Pract..

[14] Yang D., Haemmig S., Chen J., McCoy M., Cheng H.S., Zhou H., Pérez-Cremades D., Cheng X., Sun X., Haneo-Mejia J. (2022). Endothelial cell-specific deletion of a microRNA accelerates atherosclerosis. Atherosclerosis.

[15] Süren D., Yildirim M., Sayïner A., Alïkanoğlu A.S., Atalay I., Gündüz U.R., Kaya V., Gündüz Ş., Oruç M.T., Sezer C. (2017). Expression of claudin 1, 4 and 7 in thyroid neoplasms. Oncol. Lett..

[16] Saurabh D., Rajat S. (2017). Blood-Brain Barrier Permeability Is Exacerbated in Experimental Model of Hepatic Encephalopathy via MMP-9 Activation and Downregulation of Tight Junction Proteins. Mol. Neurobiol..

[17] Wu J., Gao F., Xu T., Li J., Hu Z., Wang C., Long Y., He X., Deng X., Ren D. (2019). CLDN1 induces autophagy to promote proliferation and metastasis of esophageal squamous carcinoma through AMPK/STAT1/ULK1 signaling. J. Cell. Physiol..

[18] Takasawa K., Takasawa A., Akimoto T., Magara K., Aoyama T., Kitajima H., Murakami T., Ono Y., Kyuno D., Suzuki H. (2021). Regulatory roles of claudin-1 in cell adhesion and microvilli formation. Biochem. Biophys. Res. Commun..

[19] Kim N.Y., Pyo J.S., Kang D.W., Yoo S.M. (2019). Loss of claudin-1 expression induces epithelial-mesenchymal transition through nuclear factor-κB activation in colorectal cancer. Pathol. Res. Pract..

[20] Geoffroy M., Kleinclauss A., Kuntz S., Grillier-Vuissoz I. (2020). Claudin 1 inhibits cell migration and increases intercellular adhesion in triple-negative breast cancer cell line. Mol. Biol. Rep..

[21] Chaffer C.L., Weinberg R.A. (2011). A perspective on cancer cell metastasis. Science.

[22] Lauko A., Mu Z., Gutmann D.H., Naik U.P., Lathia J.D. (2020). Junctional Adhesion Molecules in Cancer: A Paradigm for the Diverse Functions of Cell–Cell Interactions in Tumor Progression. Cancer Res..

[23] Rodrigues A.F., Fernandes P., Laske T., Castro R., Alves P.M., Genzel Y., Coroadinha A.S. (2020). Cell Bank Origin of MDCK Parental Cells Shapes Adaptation to Serum-Free Suspension Culture and Canine Adenoviral Vector Production. Int. J. Mol. Sci..

[24] Schröder M., Matischak K., Friedl P. (2004). Serum- and protein-free media formulations for the Chinese hamster ovary cell line DUKXB11. J. Biotechnol..

[25] Paillet C., Forno G., Kratje R., Etcheverrigaray M. (2009). Suspension-Vero cell cultures as a platform for viral vaccine production. Vaccine.

[26] Malm M., Saghaleyni R., Lundqvist M., Giudici M., Chotteau V., Field R., Varley P.G., Hatton D., Grassi L., Svensson T. (2020). Evolution from adherent to suspension: Systems biology of HEK293 cell line development. Sci. Rep..

[27] Dill V., Hoffmann B., Zimmer A., Beer M., Eschbaumer M. (2018). Influence of cell type and cell culture media on the propagation of foot-and-mouth disease virus with regard to vaccine quality. Virol. J..

[28] Zhang J., Qiu Z., Wang S., Liu Z., Qiao Z., Wang J., Duan K., Nian X., Ma Z., Yang X. (2023). Suspended cell lines for inactivated virus vaccine production. Expert Rev. Vaccines.

[29] Krneta-Stankic V., Corkins M.E., Paulucci-Holthauzen A. (2021). The Wnt/PCP formin Daam1 drives cell-cell adhesion during nephron development. Cell Rep..

[30] Takuya S. (2020). Regulation of the intestinal barrier by nutrients: The role of tight junctions. Anim. Sci. J..

[31] Xiao K., Song Z.-H., Jiao L.-F., Ke Y.-L., Hu C.-H. (2014). Tight junction protein levels of occludin, claudin-1 and zonula occludens-1 (ZO-1). PLoS ONE.

[32] You-Cheng H., Yu-Hsien C., Lin K.Y., Tzong-Der W., Lin H.Y., Varadharajan T., Hsin-Ling Y. (2017). Antrodia camphorata inhibits metastasis and epithelial-to-mesenchymal transition via the modulation of claudin-1 and Wnt/β-catenin signaling pathways in human colon cancer cells. J. Ethnopharmacol..

[33] Osada T., Gu Y.-H., Kanazawa M., Tsubota Y., Hawkins B.T., Spatz M., Milner R., del Zoppo G.J. (2011). Interendothelial Claudin-5 Expression Depends on Cerebral Endothelial Cell–Matrix Adhesion by β1-Integrins. J. Cereb. Blood Flow Metab..

[34] Kim D.H., Lu Q., Chen Y. (2019). Claudin-7 modulates cell-matrix adhesion that controls cell migration, invasion and attachment of human HCC827 lung cancer cells. Oncol. Lett..

[35] Fuchs M.T., Foresti M., Vielmuth F., Waschke J. (2019). Plakophilin-dependent Dsg3 oligomerization and binding properties are involved in desmosomal hyper-adhesion. FASEB J..

[36] González-Mariscal L., Miranda J., Raya-Sandino A., Domínguez-Calderón A., Cuellar-Perez F. (2017). ZO-2, a tight junction protein involved in gene expression, proliferation, apoptosis, and cell size regulation. Ann. N. Y. Acad. Sci..

[37] Yu S., Yan C., Wu W., He S., Liu M., Liu J., Yang X., Ma J., Lu Y., Jia L. (2019). RU486 Metabolite Inhibits CCN1/Cyr61 Secretion by MDA-MB-231-Endothelial Adhesion. Front. Pharmacol..

[38] Villanueva A.A., Sanchez-Gomez P., Muñoz-Palma E., Puvogel S., Casas B.S., Arriagada C., Peña-Villalobos I., Lois P., Orellana M.R., Lubieniecki F. (2021). The Netrin-1-Neogenin-1 signaling axis controls neuroblastoma cell migration via integrin-β1 and focal adhesion kinase activation. Cell Adhes. Migr..

[39] Yumi S. (2006). Possible involvement of crosstalk cell-adhesion mechanism by endometrial CD26/dipeptidyl peptidase IV and embryonal fibronectin in human blastocyst implantation. Mol. Hum. Reprod..

[40] Khademi R., Malekzadeh H., Bahrami S., Saki N., Khademi R., Villa-Diaz L.G. (2023). Regulation and Functions of α6-Integrin (CD49f) in Cancer Biology. Cancers.

[41] Resnick M.B., Konkin T., Routhier J. (2005). Claudin-1 is a strong prognostic indicator in stage II colonic cancer: A tissue microarray study. Mod. Pathol..

[42] Chao Y.-C., Pan S.-H., Yang S.-C., Yu S.-L., Che T.-F., Lin C.-W., Tsai M.-S., Chang G.-C., Wu C.-H., Wu Y.-Y. (2009). Claudin-1 is a metastasis suppressor and correlates with clinical outcome in lung adenocarcinoma. Am. J. Respir. Crit. Care Med..

[43] Morohashi S., Kusumi T., Sato F., Odagiri H., Chiba H., Yoshihara S., Hakamada K., Sasaki M., Kijima H. (2007). Decreased expression of claudin-1 correlates with recurrence status in breast cancer. Int. J. Mol. Med..

[44] Dhawan P., Singh A.B., Deane N.G., No Y., Shiou S.-R., Schmidt C., Neff J., Washington M.K., Beauchamp R.D. (2005). Claudin-1 regulates cellular transformation and metastatic behavior in colon cancer. J. Clin. Investig..

[45] Suh Y., Yoon C.-H., Kim R.-K., Lim E.-J., Oh Y.S., Hwang S.-G., An S., Yoon G., Gye M.C., Yi J.-M. (2013). Claudin-1 induces epithelial-mesenchymal transition through activation of the c-Abl-ERK signaling pathway in human liver cells. Oncogene.

[46] Oku N., Sasabe E., Ueta E. (2006). Tight junction protein claudin-1 enhances the invasive activity of oral squamous cell carcinoma cells by promoting cleavage of laminin-5 gamma2 chain via matrix metalloproteinase (MMP)-2 and membrane-type MMP-1. Cancer Res..

[47] Lemesle M., Geoffroy M., Alpy F., Tomasetto C.-L., Kuntz S., Grillier-Vuissoz I. (2022). CLDN1 Sensitizes Triple-Negative Breast Cancer Cells to Chemotherapy. Cancers.

[48] Cherradi S., Garambois V., Marines J., Andrade A.F., Fauvre A., Morand O., Fargal M., Mancouri F., Ayrolles-Torro A., Vezzo-Vié N. (2023). Improving the response to oxaliplatin by targeting chemotherapy-induced CLDN1 in resistant metastatic colorectal cancer cells. Cell Biosci..

[49] Akizuki R., Maruhashi R., Eguchi H., Kitabatake K., Tsukimoto M., Furuta T., Matsunaga T., Endo S., Ikari A. (2018). Decrease in paracellular permeability and chemosensitivity to doxorubicin by claudin-1 in spheroid culture models of human lung adenocarcinoma A549 cells. Biochim. Biophys. Acta Mol. Cell Res..

[50] Zhao Z., Li J., Jiang Y. (2017). CLDN1 increases Drug Resistance of Non-Small Cell Lung Cancer by activating Autophagy via Up-Regulation of ULK1 phosphorylation. Med. Sci. Monit..

[51] Sun N., Zhang Y., Dong J., Liu G., Liu Z., Wang J., Qiao Z., Zhang J., Duan K., Nian X. (2023). Metabolomics profiling reveals differences in proliferation between tumorigenic and non-tumorigenic Madin-Darby canine kidney (MDCK) cells. PeerJ.

[52] Li R., Li Y., Kristiansen K., Wang J. (2008). SOAP: Short oligonucleotide alignment program. Bioinformatics.

[53] Kim D., Langmead B., Salzberg S.L. (2015). HISAT: A fast spliced aligner with low memory requirements. Nat. Methods.

[54] Kim D., Langmead B., Salzberg S.L. (2012). Fast gapped-read alignment with Bowtie 2. Nat. Methods.

[55] Li B., Dewey C.N. (2011). RSEM: Accurate transcript quantification from RNA-Seq data with or without a reference genome. BMC Bioinform..

[56] Ding R., Qu Y., Wu C.H., Vijay-Shanker K. (2018). Automatic gene annotation using GO terms from cellular component domain. BMC Med. Inform. Decis. Mak..

[57] Kanehisa M., Araki M., Goto S., Hattori M., Hirakawa M., Itoh M., Katayama T., Kawashima S., Okuda S., Tokimatsu T. (2007). KEGG for linking genomes to life and the environment. Nucleic Acids Res..

[58] Kumar L., Futschik M.E. (2007). Mfuzz: A software package for soft clustering of microarray data. Bioinformation.

[59] Wang L., Feng Z., Wang X., Wang X., Zhang X. (2010). DEGseq: An R package for identifying differentially expressed genes from RNA-seq data. Bioinformatics.

[60] Langmead B., Trapnell C., Pop M., Salzberg S.L. (2009). Ultrafast and memory-efficient alignment of short DNA sequences to the human genome. Genome Biol..

[61] Nawrocki E.P., Eddy S.R. (2013). Infernal 1.1: 100-fold faster RNA homology searches. Bioinformatics.

[62] Kivioja T., Vähärautio A., Karlsson K., Bonke M., Enge M., Linnarsson S., Taipale J. (2011). Counting absolute numbers of molecules using unique molecular identifiers. Nat. Methods.

[63] Jan W.K., Marc R. (2006). RNAhybrid: MicroRNA target prediction easy, fast and flexible. Nucleic Acids Res..

[64] John B., Enright A.J., Aravin A., Tuschl T., Sander C., Marks D.S. (2004). Human MicroRNA Targets. PLoS Biol..

[65] Agarwal V., Bell G.W., Nam J.-W., Bartel D.P. (2015). Predicting effective microRNA target sites in mammalian mRNAs. eLife.

[66] Zhou Z., Lunetta K.L., Smith A.K., Wolf E.J., Stone A., A Schichman S., E McGlinchey R., Milberg W.P., Miller M.W., Logue M.W. (2019). Correction for multiple testing in candidate-gene methylation studies. Epigenomics.

