# Supplementary material for: Micro RNA-175 Targets Claudin-1 to Inhibit Madin–Darby Canine Kidney Cell Adhesion
Source: Genes (Basel). 2024 Oct 16;15(10):1333. doi: 10.3390/genes15101333 (PMC11506999; doi:10.3390/genes15101333)
Supplement: Supplementary file 1 [file genes-15-01333-s001.zip › genes-3215430-supplementary.pdf]

Supplementary data

Synthesis of gene primers

Table S1. Primer sequence information.

Primer name	species	Forward Primer(5'-3')	Reverse Primer(5'-3')
ACER2	canis	TCCCTAATATGCAGTGAAAATGCAA	AGGTGCATTTGAAGGTCCCA
ANXA9	canis	CAGACCACACAGAAGCTGGT	TCCTTTGGGTACATGGGTGC
APBB1IP	canis	GGTGAGTCCAACGACGACAT	CAGCTCTGGGTGGTTTGGAT
CLDN1	canis	CGCGGTACCCCCGTC	CAGGAAGGCCAGCAGGAAG
CYP1B1	canis	TCACCATTCCTCATGCCACC	AGAACCGGGCTGGATCAAAG
CYTH1	canis	GACGACAGCTACGTTCCCAG	CTGCCCATGGCTACCTGTTT
DDR2	canis	GGGTAGGGTTCCTGCAAACA	CAAGCCCACAAAACTGAGGC
DNAJB6	canis	GATCAGTCAGCGTAGGGACG	AGAGTTGAAAAGGAGCCGGG
DPP4	canis	GCACCTCAAAACAGCATGTCA	TTGCGAACACAGAAGTCCCT
DSG3	canis	TCAAGGAGCTTCCGCTTTGT	GGTTGCACGAGAGAACCAGA
FOCAD	canis	AGTGGCACCGTGCAATTTTC	AGTTGCAAGTCCAGCTCTCC
HAS2	canis	GTGGAGTGCGAATGTGGAGA	GGCATGCTGTGTGTTTCGTT
ITGA6	canis	GTGCATAACCGACCAGGGAA	CTGGGGAAGGGCAAAGAGAG
ITGB8	canis	AAGCGTTTGCACAAGAGCAG	AAGTGGACAACCAGCAACCA
ITPKB	canis	TTGCTATGGCAAGGTCTGGG	CCCCAATCTGCTGGGGATTT
MOG	canis	TGCCATGTCGCATCTATCCC	TTACCTTCCCCTCGCCCATA
NID2	canis	ACAGCGGCCAGTTTACTGAT	ATTTACTTTCTTCCTGCTGGGC
NPNT	canis	CCAGGATAAGGTGCCAGCTC	TGTGGCTTGATGATCCAGGG
NTN1	canis	CCTGCAAGTGCCCCAAAATC	ATCACCAGGCTGCTCTTGTC
ONECUT1	canis	AGCACAGGTCAGCAATGGAA	CCGGACTTGAGTTTGCTCCA
PLPP3	canis	TCTTGAACGTCTGCAACCCT	GGACACAAAGAAAACCACCAA
PSEN1	canis	TGGCGGCAAGTGAAGAGTAG	GGGGTGCAGGTAACTCTGTC
TJP2	canis	AGCTGTCCGAACACTCGAAG	GTTCAGGTCCCACATCTCCG
VAPA	canis	GCCCACCGACCTCAAATTCA	TAGGCCTCACACAGTACCGA
VAV1	canis	GGCCAGGGCCATAGCAATAA	GTTCCTAGCACCTGGCATGA
WDPCP	canis	AATGACAGCCCAGAATGGCA	ACCACAGGTGCAGTTCAGTC
ARFIP1	canis	CGTGATGCAAACACTCTGCC	CAGCAATGGCGTTGTGGAAA
BCAS3	canis	TTGTGGGATCCGGAACAGGG	CCATCCCGAACACCTGCTTT
FLRT2	canis	CTGGAAACGCACGTGAACAA	CCTACCGGGGAGGGAACTAA
SNAI2	canis	GGACTCTTTAGGTCGCTCGG	CGGTAATGGGGCTGTATGCT
ERBB3	canis	GGGGGTAGAGAATGTGTCCC	GAGCACGATCTCGAGGTTCC
STAB2	canis	GCGGACATAGGAAAAGCCCT	ATGATTCCAACGACGCCAGA
EPHA1	canis	ACATTCTGCACTTCCGCTCA	GGGCTCAGTCCTTGAATCCC
SMAGP	canis	GTTTCTGTGGCTGCCCAATG	CACTAGTTGACCAGGCAGGG
AKAP12	canis	GATGCCCTGGTGGAGTAACC	CTCCTCCTGCCCATCCTTTG
EFNA5	canis	CTGTGAAACAACCCCCAGGA	GAGCCCTGTCTTGGGGAAAA
LIMS2	canis	AAGGAGGTGGAGTGGTGACT	GAAGAGCCCCTCAGGGAATG
DENND6A	canis	AGTCATCTTAGCTGTGGGCA	CGAAATGGAATGCGGGGACT

VPS33B	canis	ATCGGATCGCCAATGTCTCC	CGCATGTTCTTGATGCGAGG
BCAS3	canis	TTGTGGGATCCGGAACAGGG	CCATCCCGAACACCTGCTTT
STX16	canis	CCAGTTAGTGCTGGTGGAGC	ATCAGGACCCAAATCCCTGC
APBB1IP	canis	GGTGAGTCCAACGACGACAT	CAGCTCTGGGTGGTTTGGAT
AKAP12	canis	GATGCCCTGGTGGAGTAACC	CTCCTCCTGCCCATCCTTTG
TNS2	canis	GGAATGTTCCAGGCAGCAGG	AGTCCCCTCACCTGGTAGTT
CNN3	canis	GAACAGGAACGAACGGGTCT	TCGCCGTATTGGTAATCCCG
SPINT2	canis	GTGCCGGGGCAATAAAAACA	GGACCACCACTTTAGTGCCA
LYPD3	canis	TGACTGCAGCGAATGTGACT	CAGGACAAGGGGTGGGATTC
GCNT2	canis	AAACAGCTCTTGGGGCAACT	GATTCCTTTGCTGAAGCGGC
GRB7	canis	AAAGTCGGGACACCCTCCTA	GGGTCCGAAGTCTGGTTCTG
TEK	canis	GGCCCAAGCCTTCCAAAATG	TCCTTCTTGATGCGTGCCTT
miR-175	canis	TGGCAGCCGAGCCCCGAC	AGTGCAGGGTCCGAGGTATT
miR-123	canis	TGCCCCGCCGGGTCGGCCG	AGTGCAGGGTCCGAGGTATT
miR-371	canis	ACTCAAAAAATGGCGGCACTTT	AGTGCAGGGTCCGAGGTATT
miR-172	canis	GCGCCCGGGGAAGCTAGCCT	AGTGCAGGGTCCGAGGTATT
U6	canis	CTCGCTTCGGCAGCACA	AGTGCAGGGTCCGAGGTATT
GAPDH	canis	GAACATCATCCCTGCTTCCA	CAGGTCAGATCCACAACTGATAC

Table S2. All the valid URLs of the bioinformatics analysis software.

Software	URL
SOAPnuke(1.5.0)	https://github.com/BGI-flexlab/SOAPnuke
cmsearch (1.1.2)	https://omictools.com/cmsearch-tool
miRDeep2 (0.1.3)	https://github.com/rajewsky-lab/mirdeep2
Piano	http://ento.njau.edu.cn/Piano.html
DEGSeq(v1.36.0)	http://bioinfo.au.tsinghua.edu.cn/software/degseq/
RNAhybrid(0.1)	https://bibiserv.cebitec.uni-bielefeld.de/rnahybrid
miRanda (V22)	http://www.microrna.org/microrna/home.do
TargetScan (7.1)	http://www.targetscan.org/vert_72/
Mfuzz(2.60.0)	http://www.bioconductor.org/packages/release/bioc/html/Mfuzz.html
HISAT2(v2.2.1)	http://www.ccb.jhu.edu/software/hisat
Bowtie2 (v2.4.5)	http://bowtie-bio.sourceforge.net/Bowtie2/index.shtml
RSEM (v1.3.1)	http://deweylab.biostat.wisc.edu/rsem/rsem-calculate-expression.html
GO	http://geneontology.org
KEGG	http://www.kegg.jp/kegg/rest/keggapi.html
WGCNA (v1.71)	https://labs.genetics.ucla.edu/horvath/CoexpressionNetwork/Rpackages/WGCNA
